# High genetic merit dairy heifers grazing low quality forage had similar weight gain and urinary nitrogen excretion to those of low genetic merit heifers

**DOI:** 10.3389/fvets.2023.1234872

**Published:** 2023-10-06

**Authors:** L. Cheng, C. L. Goulven, B. R. Cullen, C. Clark, P. Gregorini, X. Z. Sun, S. Talukder

**Affiliations:** ^1^Faculty of Science, School of Food, Agriculture and Ecosystems Science, University of Melbourne, Parkville, VIC, Australia; ^2^Higher Institute for Agricultural Sciences, Food Industry, Horticulture and Land Management Agrocampus Ouest, Rennes, France; ^3^School of Life and Environmental Sciences, Faculty of Science, The University of Sydney, Sydney, NSW, Australia; ^4^Department of Agricultural Sciences, Faculty of Agriculture and Life Sciences, Lincoln University, Canterbury, New Zealand; ^5^The Innovation Centre of Ruminant Precision Nutrition and Smart Farming, Jilin Agricultural Science and Technology University, Jilin, China; ^6^AgResearch Ltd., Hamilton, Waikato, New Zealand

**Keywords:** heifer, genetic merit, production, low quality forage, environmental pollution dairy heifer, forage, environment, excretion

## Abstract

Climate variability and increasing drought events have become significant concerns in recent years. However, there is limited published research on body weight (BW) change of dairy heifers with different genetic merit when grazing on drought impacted pastures in southern Australia. Achieving target body weight (BW) is vital for dairy heifers, especially during critical stages like mating and calving. This study aimed to assess dry matter (DM) intake, BW change, urinary nitrogen excretion, and grazing behaviours of high vs. low genetic dairy heifers grazing pasture during a 43-day experimental period in a drought season. Forty-eight Holstein Friesian heifers grazed on ryegrass-dominant pasture and were divided into two groups based on their high and low Balanced Performance Index (HBPI and LBPI, respectively). Each group was further stratified into six plots, with similar BW, resulting in four heifers per replication group. Data from the five measurement days were averaged for individual cows to analyse the dry matter intake, nitrogen intake and nitrogen excretion. The statistical model included the treatment effect of BPI (H and L) and means were analysed using ANOVA. The pasture quality was poor, with metabolizable energy 9.3 MJ/Kg DM and crude protein 5.9% on a DM basis. Nitrogen intake and urinary nitrogen excretion were significantly higher (*p*  <  0.05) in HBPI compared to the LBPI. However, despite these differences, the study did not find any advantages of having HBPI heifer grazing on low quality forage in terms of BW performance.

## 1. Introduction

Pasture containing a mixture of ryegrass and clover are the main feed in Australasian pasture-based dairy production systems (1). Without irrigation, livestock grazing on such pasture mixtures often encounter challenges like low and variable herbage growth in summer and autumn and poor quality in late spring and summer (2). In recent years, the increasing climate variability, characterised by shorter spring and more frequent drought events, has led to fluctuations in plant growth and biomass production in pasture-based dairy systems, resulting in lower utilisation of grazed pastures (3). The combination of warmer conditions and low soil moisture further contributes to the production of low-quality forage, significantly reducing nutrient availability (e.g., leaf protein content) and digestibility (4, 5). Animals grazing these low-quality forages face challenges in meeting their required dry matter intake (DMI) to achieve their production targets.

In Australia, the genetic merit of a dairy cow is measured using Australian Breeding Values, which take into account traits influencing milk production, reproductive efficiency and feed efficiency.^1^ The Australian national breeding objective, known as the Balanced Performance Index (BPI), includes traits that contribute to cow profitability, farmer preferences and desired gains (6). Breeding Indices consolidate multiple traits into a single value for comprehensive evaluation. The Balanced Performance Index serves as an economic indicator that stimulates enhancements in traits influencing lifetime contribution to production, type, health, fertility, longevity, workability, feed efficiency, and type (see text footnote 1). This aligns harmoniously with the preferences of Australian farmers, as identified in the National Breeding objective review (7).

To fully express their genetic potential, animals should be given optimal nutrition. In pasture-based systems, cows obtain a significant portion of their daily and annual rations from grazing (8). Much of the management of pasture-based dairies is dictated by climate. Climate change scenarios for South-Eastern Australia indicate increasing temperatures, declining rainfall, and longer dry summer seasons (9), which may negatively impact regional pasture-based systems (10). A research gap exists concerning the production potential of high genetic merit heifers grazing low-quality pastures.

Environmental pollution mitigation is a major focus for sustainable dairying. Nitrogen (N) ingested from the pasture can be excreted through manure and transformed into various ions or gaseous forms, contributing to global warming (11). Additionally, N in urine patches deposited into the soil can lead to nitrate leaching and pollute groundwater and waterways (12). Previous work (13) showed that high genetic merit cows offered moderate quality pasture (metabolisable energy = 9.9 MJ/Kg DM, crude protein = 15.2% on DM basis) had a lower urinary N excretion and higher N use efficiency (milk N excretion/N intake) compared to those with lower genetic merit. Hence, the hypothesis of this study was that higher genetic merit dairy cattle grazing drought impacted pasture would have a lower environmental pollution impact (e.g., urinary N excretion). Therefore, the objective of this study was to quantify urinary N excretion of high vs. low genetic dairy cattle grazing pasture during drought season.

## 2. Materials and methods

### 2.1. Ethical statement

This study utilised dairy heifers sourced from the Dookie Campus, The University of Melbourne, Australia. The research protocol received approval from The University of Melbourne Animal Ethics Committee (#1814440.1).

### 2.2. Paddock and forage preparation

A 6.5-ha paddock was selected for the study and sown with a mixture of ryegrass and clover. The mixture comprised Italian ryegrass (*Lolium multiflorum*, cv. Crusader, 10 kg seed/ha), hybrid ryegrass (*Lolium boucheanum*), tetraploid ryegrass (*L. multiflorum x L. boucheanum,* cv. Zoom, 10 kg seed/ha), Persian clover (*Trifolium resupinatum*, cv. Shaftal, 3 kg seed/ha) and balancia clover (*Trifolium michelianum*, cv. Viper, 2 kg seed/ha). Glyphosate (2 L/ha) was sprayed on the paddock 3 days before sowing, using a 2006 3,000 L 30mt boom spray (Hardi, Australia). Monoammonium Phosphate fertiliser applied at a rate of 60 kg/ha using a seeder (Aitchison 3132ct, Australia), equipped with roller and baker boot tyres.

### 2.3. Grazing study design

Before commencing the grazing study, the paddock was divided into 12 replication plots. Six plots were assigned for grazing heifers of high Balanced Performance Index (BPI; HBPI), and six plots were assigned to grazing heifers with low BPI (LBPI). Each treatment had a spatial six replication groups. The study was conducted from September 13 to October 26, 2018, with a 14-day adaptation period followed by a 29-day measurement period. Throughout the study period, daily temperature ranged from 5.9 to 21.2°C and relative humidity ranged from 38.6 to 91.9%.

A total of 65 Holstein Friesian heifers, aged between 12 and 19 months and born between March and October 2017, were sourced and genotyped for BPI.^2^ The Balanced Performance Index (BPI) functions as an economic gauge, encouraging improvements in attributes that impact lifetime contributions to production, type, health, fertility, longevity, workability, feed efficiency, and type. Prior to the study, two BPI groups were formed, consisting of 24 HBPI heifers and 24 LBPI heifers. Each HBPI and LBPI group was further subdivided into six plots with similar body weight (BW) resulting in four heifers per replication group. All heifer replication groups had free access to a water trough.

### 2.4. Forage allocation

The heifers were managed under a set stocking grazing system. To determine the appropriate forage allowance for each heifer, the metabolisable energy (ME) requirements for maintenance along with a 0.8 kg daily body weight gain [BWG (14);] per heifer were considered. Forage ME content was assumed to be 10 MJ ME/kg DM based on previous pasture quality data from the region. The individual heifer forage allowance (kg DM/heifer per day) was calculated using the following formula:


$$\begin{array}{l} \mathrm{Heifer}\;\mathrm{forage}\;\mathrm{allowance}=[0.65\times BW{\left(\mathrm{kg}\right)}^{0.75}\\ +\;40\;\left(MJ\;\mathrm{ME}/\mathrm{heifer}/\mathrm{day}\right)]÷10\;\left(MJ\;\mathrm{ME}/\mathrm{kgDM}\right) \end{array}$$

The group forage allowance was obtained by multiplying the individual heifer forage allowance by the number of heifers in each replication group and the expected number of grazing days. Pre-grazing forage mass was estimated by a calibrated ruler, while post-grazing forage mass was assumed to be 800 kg DM/ha per farm operation standard (Gabler, personal communication).

### 2.5. Forage quality measurements

Forage quality and botanical composition samples were collected by cutting 40 samples of 0.01 m^2^ randomly to ground level per replication plot on measurement days −3, 9, 15, and 27. These samples were bulked per replication plot and used to estimate the dry matter percentage (DM%), chemical composition, and botanical composition. The samples were divided into two subsamples. One subsample was weighed fresh and then oven-dried at 65° C for 48 h to determine DM%. The oven-dried pasture samples were then ground to 1 mm for analysis of nutritive value by the New South Wales Department of Primary Industries (Australia) Laboratory Services using near-infrared reflectance spectrophotometry (Foss NIRSystems 5000, FOSS NIRSystems Inc., United States). This analysis estimated N, crude protein (CP; N × 6.25), digestible organic matter content in the dry matter, and neutral detergent fibre (NDF). The second subsample was separated into ryegrass, clover, weed and dead matter before oven drying at 65°C for 48 h and weighing to determine botanical composition on a DM basis.

### 2.6. Heifer measurement

The apparent DMI of each replication group was calculated based on the difference between pre- and post-grazing forage mass, forage regrowth rate and area grazed. The pre and post-grazing forage mass for each replication plot was estimated using a calibrated rising plate metre (RPM; EC09 RPM—Jenquip, Fielding, New Zealand). The relationship between the compressed height of RPM and forage mass was established by cutting 30 quadrats, each 0.1 m^2^, to ground level. Forage samples were weighed fresh, oven-dried at 65°C for 48 h to determine DM% and calculate herbage DM yield/ha. Linear regression was used to establish the relationship between post-grazing forage mass and RPM compressed height.

All heifers were weighed after a 12-h fasting on measurement days 0 and 29. Body size and body length were also recorded on days 0 and 29, using a graduated tape (WI 53190, The Coburn Company, Inc., Whitewater, United States). The body length was measured as the distance between the withers and the tail head, and the body size was measured as heart girth following the Coburn Company protocol (15).

On measurement days 16 and 25, the heifers were herded into the cattle yards for urine and blood sampling. Mid-stream urine samples were collected following vulva stimulation and immediately acidified to a pH below 3.0 using concentrated sulphuric acid (50% v/v) to prevent ammonia volatilisation. Blood samples were collected from the coccygeal vein utilising sodium heparin vacuette tubes (BD Vacutainer, Belliver Industrial Estate, United Kingdom). Subsequently, the blood underwent centrifugation at 3,000 *g* and 4°C for a duration of 15 min to extract plasma. Both urine and plasma samples were stored at −20°C until analysis. Urinary N concentration was determined using an N analyser (Vario MAX CN, Elementar Analysensysteme, Hanau, Germany). Plasma urea N (PUN) was analysed using a Cobas Integra 400 plus Daytona RX Clinical Analyser (RocheRandox, Nishinomiya, Japan, Switzerland). Urinary N excretion (UN) during the measurement period was estimated from plasma (PUN) and average BW using the equation published by Kohn et al. (16): UN (g N/day) = 1.3 × PUN (g/L) × average LB (kg). The BW value used in these calculations was the average BW of the start and end of the measurement period.

Heifer grazing time was monitored according to the method described by Cheng et al. (17). On measurement days 23 and 29, grazing time was recorded at five intervals during a 24-h grazing period: between 6:15 and 7:15, between 12:15 and 13:15, between 15:15 and 16:15, between 18:15 and 19:15, and between 19:15 and 19:55. In addition, on measurement days 23 and 29, bite rate of four heifers from each replication group was also recorded for an hour (6.15–7.15) each day.

### 2.7. Statistical analyses

The GENSTAT statistical package (version 16, VSN International Ltd., Hemel Hempstead, United Kingdom) was used for comprehensive ANOVA and linear regression. The statistical model incorporated the treatment effect of BPI (H and L). For each variable, data from the five measurement days were aggregated on an individual cow basis, and means were analysed using ANOVA. The significance of the treatment effect was considered at a threshold of *p* < 0.05.

## 3. Results

During the study period, heifers grazed with ryegrass-dominant pasture. On a DM basis in the sward, the percentages of ryegrass, white clover, weed, and dead matter in HBPI were 59.8, 0.7, 1.0, and 38.5%, while those were 59.0, 0.5, 2.0, and 38.5% in LBPI group, respectively. The ME content was 9.23 and 9.25 MJ/kg DM, and N% was 1.25 and 1.1% in HBPI and LBPI, respectively (Table 1). Apparent DMI, heifer BW, body length and size did not differ significantly (*p* > 0.05) between two treatments groups. However, urinary N excretion was 24% higher in the HBPI group (*p* < 0.05; Table 2) compared to the LBPI group. Apparent N intake and plasma urea N tended to be higher in the HBPI group compared to the LBPI group (*p* = 0.05 and 0.06, respectively).

**Table 1:** Dry matter content and nutritive value of forage wheat grazed by heifers in high and low balanced performance index (HBPI and LBPI, respectively) groups.

Parameters	HBPI	LBPI
DM (%)	41.6	40.5
ME (MJ/kg DM)	9.2	9.3
NDF (% DM)	57.7	57.8
CP (% DM)	7.7	7.1
N (% DM)	1.3	1.1
Ash (% DM)	7.0	7.1
OM (% DM)	93.0	92.0
DMD (% DM)	65.5	65.9
OMD (% DM)	64.6	63.9

DM, Dry matter; ME, Metabolizable energy; NDF, Neutral detergent fibre; CP, Crude protein; N, Nitrogen; OM, Organic matter; DMD, Dry matter digestibility; OMD, Organic matter digestibility.

**Table 2:** Body weight at the start and end of the study, body length, body size, average daily growth, apparent dry matter intake, plasma urea nitrogen, urinary nitrogen percentage, and nitrogen loading and grazing behaviour in high and low balanced performance index (HBPI and LBPI, respectively) groups.

Parameters	HBPI	LBPI	SED	*p* value
Average daily growth (kg/heifer/day)	1.2	1.1	0.21	0.66
Body weight at the start of study (kg/heifer)	369.5	339.9	22.7	0.02
Body weight at the end of study (kg/heifer)	408.6	372.6	41.4	0.35
Body length at the start of study (cm/heifer)	115.7	111.7	15.3	0.47
Body length at the end of study (cm/heifer)	115.7	112.3	4.5	0.06
Body size at the start of study (cm/heifer)	175.0	165.4	9.4	0.81
Body size at the end of study (cm/heifer)	180.0	173.4	4.5	0.95
Apparent dry matter intake (kg DM/heifer/day)	11.5	10.8	0.91	0.60
Metabolizable energy intake (MJ/day/heifer)	106.2	100.0	7.84	0.45
Feed conversion efficiency	0.102	0.108	0.02	0.79
Apparent nitrogen intake (g/cow/day)	111.9	95.4	7.53	0.05
Urinary nitrogen excretion (g/day/heifer)	42.9	34.5	3.50	0.03
Plasma urea (mmol/L)	1.96	1.45	0.25	0.06
Urinary nitrogen (%)	0.17	0.11	0.05	0.26
Grazing time (min/5 h observed)	89	91	4.7	0.55
Bite rate (bites/min)	37.8	39	1.51	0.45

SED, Standard error of differences.

## 4. Discussion

The objective of this study was to determine the impact of genetic merit on BW performance and urinary N excretion in heifer groups grazing on a pasture during a drought season. The BW did not differ between LBPI and HBPI groups. The apparent DMI of heifers in the current study aligned with the previous studies, falling within the range of 2.5–3% of heifer BW (17–19). The insignificant differences in DMI, grazing time and bite rate indicate that grazing conditions were similar between genetic merit groups. The lack of BW differences between genetic merit groups could potentially be attributed to two factors. First, both groups achieved similar ME intake and feed conversion efficiency. Second, inadequate CP supply might have limited the HBPI group from fully expressing their genetic potential for better growth. The CP content of the grazed pasture was 7.4% on DM basis, substantially lower than growing heifer’s requirement [14% on DM basis (20)]. Further to note that the pasture contained on average 41.1% DM, 57.7% NDF, 9.3 MJ ME/kg DM and 38.5% dead matter on DM basis, reflecting the quality of pasture is poor, is likely due to drought effect led to matured pasture and reduced nutritive value (21).

Due to climate change, Northern Victoria, Australia is facing significant challenges, including higher spring temperatures, more frequent heatwaves, reduced cool season rainfall, prolonged droughts, limited water availability (22). These changes in climate have implications for the dry matter production and nutritive characteristics of ryegrass-based pasture in the region (23). It is most likely that the percentage of CP in grazed pasture was a limiting factor in this study. Particularly in high genetic merit animals, pasture with lower nutritional value might cause a hindrance in expressing the animals’ full genetic potential as an environmental effect.

Despite no significant differences in BW between LBPI and HBPI groups, the study revealed that urinary N excretion was higher in HBPI compared to LBPI, indicating that HBPI heifers could potentially cause higher N pollution in the current dryland grazing system (24). The higher N intake was likely to be the main reason led to a 24% higher urinary N excretion in HBPI compared to LBPI, with no nitrogen use efficiency difference being detected in two groups (i.e., PUN as an indicator of nitrogen use efficiency).

The lower pasture quality could indeed pose a limitation to the study, as it may hinder the heifers’ access to adequate nutrients, potentially impacting their growth and ability to fully express their genetic potential. To address this limitation and obtain more comprehensive insights, a controlled study should be conducted, involving heifers with both low and high genetic potential and providing them with pastures of varying qualities. To enhance the study’s robustness, it is essential to increase the number of animals participating in the research and extend the measurement period. By doing so, the statistical power of the study will be improved, allowing for more reliable conclusions about the genetic effects on feed conversion and weight gain in heifers. Additionally, the extended measurement period will enable a more thorough examination of the long-term impacts of genetics and pasture quality on the animals’ growth and development.

Furthermore, measuring the body condition score of the heifers would be a valuable addition to the study. Body condition scoring can help assess the overall health and nutritional status of the animals, providing essential information about how genetics and feed quality interact to influence their growth and development. This additional metric would contribute to a more comprehensive understanding of the relationship between genetics, feed quality, and the expression of genetic potential in heifers.

### 4.1. Practical implications

The heifers’ ability to convert feed into body weight can be significantly influenced by the genetic potential of the stock. Heifers with a high growth potential tend to consume more feed and gain more weight from the same amount of feed when compared to heifers with lower growth potential. This effect is particularly pronounced in animals with high genetic merit. However, it is important to note that the environment also plays a crucial role in shaping the expression of their genetic potential. When these high genetic merit animals are exposed to pastures with lower nutritional value, the environmental conditions may act as a hindrance, preventing them from fully expressing their genetic potential. In such cases, the limitations imposed by the lower nutritional value of the pasture can impact their growth and weight gain, despite their inherent genetic advantages.

## 5. Conclusion

The results indicated that apparent DMI, body weight, grazing time, and bite rate were similar between the HBPI and LBPI groups. This suggests that genetic merit did not significantly impact these aspects of heifer performance under the given conditions. However, the HBPI group showed higher N intake and urinary N excretion compared to the LBPI group. This indicates that genetic merit might play a role in the heifers’ nitrogen metabolism when exposed to low-quality pasture during drought conditions. Overall, the study did not observe any clear advantages of having HBPI heifers grazing on low-quality forage in terms of body weight performance. Nonetheless, the higher N intake and urinary N excretion in the HBPI group warrant further investigation and could potentially have implications for nutrient management strategies in such conditions.

## Data availability statement

The original contributions presented in the study are included in the article/supplementary material, further inquiries can be directed to the corresponding author.

## Ethics statement

The study was undertaken with the approval of The University of Melbourne Animal Ethics Committee (#1814440.1).

## Author contributions

LC: research design, conceptualisation, methodology, and writing draft. CG: field work, data analysis, and writing draft. BC, CC, PG, and XS: conceptualisation and feedback on draft. ST: data analysis and draft preparation. All authors contributed to the article and approved the submitted version.

## Footnotes

^1^https://datagene.com.au

^2^DataGene.com
